# “Looking over the Backyard Fence”: Householders and Mosquito Control

**DOI:** 10.3390/ijerph14030246

**Published:** 2017-03-02

**Authors:** Samir Mainali, Ram Sharan Lamichhane, Kim Clark, Shelley Beatty, Maria Fatouros, Peter Neville, Jacques Oosthuizen

**Affiliations:** 1 School of Medical and Health Sciences, Edith Cowan University, Joondalup, WA 6027, Australia; smainal0@our.ecu.edu.au (S.M.); rlamichh@our.ecu.edu.au (R.S.L.); k.clark@ecu.edu.au (K.C.); peter.neville@health.wa.gov.au (P.N.); j.oosthuizen@ecu.edu.au (J.O.); 2 Town of Bassendean, Perth, WA 6054, Australia; mfatouros@bassendean.wa.gov.au; 3 Health Department of Western Australia, Perth, WA 6000, Australia

**Keywords:** mosquito(es), vector-borne disease, *Aedes notoscriptus*, *Aedes camptorhynchus*, *Aedes vigilax* container-inhabiting mosquitoes, public health

## Abstract

(1) Background: Vector-borne diseases are a significant public health problem in Western Australia. Mosquitoes are responsible for the transmission of a number of pathogens and may pose a serious nuisance problem. Prevention efforts in the State are multi-faceted and include physical, chemical, and cultural control methods for restricting mosquito breeding. This is less complex where breeding areas are located within public open spaces. In Australia’s developed urban areas, breeding sites are, however, frequently located within private residential landholdings, where the scope of public health officials to act is constrained by law and practicality. Consequently, mosquito prevention in these locations is predominantly the responsibility of the residents. This research addressed a gap, both in understanding the degree to which “backyard” mosquito breeding has the potential to contribute to local mosquito problems, and in assessing what residents “think and do” about mosquito control within their home environment. (2) Methods: The study was conducted in the Town of Bassendean, a metropolitan Local Government Area of Perth, Western Australia, in close proximity to two natural, productive mosquito breeding sites, namely Ashfield Flats and Bindaring Park. A total of 150 householders were randomly surveyed during the summer of 2015–2016, to gauge residents’ knowledge, attitudes, and practices (KAP (knowledge, attitudes, and practices) Survey) in regards to mosquitoes, their breeding and ecology, and avoidance or minimization strategies. The survey comprised nine questions covering residents’ knowledge (3 questions), attitudes (3 questions), and practices (3 questions), as well as additional questions regarding the basic demographics of the resident. Larvae were collected from backyard containers and reared to adults for species identification. A series of Encephalitis Vector Surveillance carbon dioxide (EVS CO_2_) traps were also deployed, to assess adult mosquito density and species composition. (3) Results: *Aedes notoscriptus* (Skuse), a known container-inhabiting species, accounted for just over 50% of all mosquitoes identified. Most residents were aware of mosquito-borne disease and its risk in their local area. While the majority (79%) of the sample correctly identified Ross River virus as the most common infection in WA, a significant gap in the general knowledge of residents in regards to mosquito biology and breeding habits, was noted. Furthermore, only 50% of residents reported using personal protective measures to reduce mosquito bites and only one in six residents undertook physical or chemical mosquito control around their home. Additionally, 60% of respondents believed that mosquito control was “a job for the council and the state government”, rather than for individual householders. (4) Conclusions: A significant gap in the knowledge of residents in the study area existed in regards to the general knowledge of mosquitoes and their breeding habits; types of treatments that could be employed within the home; and the residents’ responsibility for the management of mosquito breeding on their private property. A public education campaign has been deployed to educate the residents.

## 1. Introduction

Vector-borne infections are responsible for 17% of the global burden of disease [1]. This equates to approximately one billion cases of vector-related disease annually, resulting in one million deaths [1]. Across Western Australia, endemic vector-borne pathogens include Ross River virus (RRV) and Barmah Forest virus (BFV); while Murray Valley encephalitis (MVEV) and West Nile virus (Kunjin substrain) (WNV_KUN_) are only found in the northern two thirds of the State [2]. Additionally, returning overseas travelers are often diagnosed with mosquito-borne diseases, including dengue (DEN), chikungunya (CHIK), and malaria [2]. In each case, mosquitoes are the vector responsible for the transmission of the virus to humans.

In 2016, almost 10,000 Australian cases of RRV were recorded; more than two-in-three vector-borne infection cases in this country [2]. In Western Australia, where the current study was undertaken, RRV is also the most commonly-reported vector-borne infection, with almost 1000 cases reported in 2016, equating to one-in-ten of the national cases and corresponding to a risk that is consistent with that experienced across the country [2]. RRV is a non-fatal mosquito-borne virus with symptoms that include nausea, a rash, fever, polyarthritis, and arthralgia [3,4].

Mosquito populations increase when environmental conditions are favorable. Among the determinants of this are broader climatic conditions that are beyond human control and those that are wholly or partially preventable. Because human behavior is potentially modifiable, it is critical to understand this aspect and its determinants (e.g., people’s knowledge and attitudes), in relation to elements that might foster mosquito breeding. This is especially true because behavior has been found to be predictive of the prevalence and range of some mosquito-borne infections [5]. In urban neighborhoods, like the location of the current study, behaviors of particular concern center on the propensity of householders to leave containers capable of collecting water in outdoor areas, because these can provide a habitat for species like *Aedes notoscriptus* [6]; known vectors of RRV and BFV [7]. Further, studies by Trewin et al. [7] indicated that water-hoarding behaviors associated with a warmer, drier climate led to an increase in the number of water-holding containers within residential lots of Brisbane, Australia.

In Western Australia’s capital, Perth, urban sprawl has led to an increasing number of settlements located close to areas known to be sites of high mosquito breeding, and these have been strongly linked in terms of the spatial distribution of RRV in the metropolitan area [8]. *Aedes camptorhynchus* (Thomson) and *Aedes vigilax* (Skuse) are both known to breed in the saltmarshes along the Swan River, often in large numbers under suitable environmental conditions, and are in close proximity to the residents of the current study. In Perth, *Aedes vigilax* is considered to be an aggressive biter during the hottest months of the year, and will feed on humans and other hosts during the day and night [9]. Local Government mosquito management programs are largely focused on controlling saltmarsh species, due to their aggressive nature and infection risk, and the ability to treat natural breeding sites through physical and chemical control methods. However, gaining access to private residential lots can be difficult and requires the permission of the landholder, prior to entry. Thus, the contribution to the mosquito fauna from backyard containers (particularly species like *Aedes notoscriptus*) remains largely unknown.

Thus, residents living in these areas have an elevated risk of contracting mosquito-borne infections, in addition to the increased nuisance impact [10]. Consequently, it is necessary to develop an understanding of the knowledge base of this exposed sub-group of the population regarding the management of mosquito-rearing containers in their own backyards, and their approaches to broader personal and household preventive practice. This information can then be used to design effective public health education programs. Bodner [11] argues that backyards are “private spaces”, and public health intervention directed at mosquito breeding in these areas is generally restricted to the deployment of public health education campaigns. In the study location, mosquito management in open space, including the river foreshore, is the responsibility of the Local Government; however, the local health laws require the residents to keep premises “free of mosquito breeding matter” [12].

Despite the importance of the household-level knowledge, attitudes, and practices (i.e., behavior-related dimensions) regarding mosquito-borne infection risk and the magnitude of Western Australian health problems caused by these vectors, local research on the issue has been scant. In what appears to be the only prior behavior-related study undertaken on mosquitoes in the State [13], a phone survey found the householder knowledge of backyard breeding to be limited, with only one-in-five respondents (21%) aware that mosquitoes can breed in backyard containers.

It is somewhat paradoxical, therefore, that while both the State Department of Health and metropolitan local authorities implement active mosquito control efforts in open-space areas known to be significant mosquito breeding sites, what is happening in nearby residential backyards remains unknown. This is a significant gap because it doesn’t allow tailored, accurate public health messages to be given to households about the contribution that their own control efforts might make and the extent to which additional efforts might diminish their experience of mosquito nuisance and infection, and subsequent disease risk.

The current study, undertaken in metropolitan Perth, was an attempt to address the previously-mentioned gap. Along with quantifying householder knowledge, attitudes, and practices (KAP) in relation to mosquito nuisance and control, the study determined the frequency and range of potential backyard breeding sites in a selected geographic area known to be at high risk of intense mosquito nuisance problems, to determine whether backyard-inhabiting species were common. This study was intended to contribute to our understanding of residential-level causes of mosquito problems in urban areas, and to aid with the development of tailored household education on managing mosquitoes.

## 2. Materials and Methods

The research was undertaken between December 2015 and March 2016, spanning the period which is historically associated with the greatest burden of mosquito nuisance in Perth. The study area comprised approximately seven square kilometers of medium-density housing and parkland, located in the Town of Bassendean on the Swan River tidal inundation zone, approximately 12 km from the Perth Central Business District. It is an area known to contain two highly problematic mosquito breeding sites, located in separate public open space areas including Ashfield Flats, a 400 m^2^ saltmarsh tidal breeding site on the edge of the Swan River, and Bindaring Park, a brackish creek running through the suburb to the Swan River. Mosquito nuisance and infection risk in the study area are usually attributed to the presence of two saltmarsh breeding mosquito species (*Aedes camptorhynchus* and *Aedes vigilax*) that can dominate the mosquito fauna after tidal inundation of the Ashfield Flats site. Reflecting its status as a mosquito-problem-area, the local government has implemented an extensive mosquito control effort on public land, to reduce the impact of these two saltmarsh species on nearby residents. For example, the Bassendean council places approximately 400 s-methoprene briquettes across Ashfield Flats each year, in order to control mosquito numbers. However, the extent of backyard breeding (including the role of *Aedes notoscriptus*) has not been quantified within the Perth Region.

Abutting the known public open space breeding sites is extensive medium-density residential housing, lying at distances spanning as little as 50 m through to more than one kilometer, from known mosquito breeding sites. Two postgraduate public health students of Edith Cowan University (ECU) undertook most of the field data collection during their summer vacation, under supervision and with the support of a range of people, including a senior medical entomologist employed by the State Department of Health, an environmental health officer employed by the relevant local government authority, and academic staff with experience in both behavioral research and mosquito control studies. Both students had prior undergraduate training; one in environmental health and the other in general medicine. Prior to undertaking their field work for the current study, both students completed a week-long specialist mosquito management course, to extend their knowledge and skills in relation to mosquito trapping and identification. During the early phase of field work, quality control was guaranteed by ensuring that the students were accompanied by the medical entomologist. Field work included obtaining permission and administration of a householder knowledge, attitude, and practices questionnaire, as approved by the Edith Cowan University’s Human Ethics Research Committee (13843).

### 2.1. Study Area Segmentation

**Figure 1:**
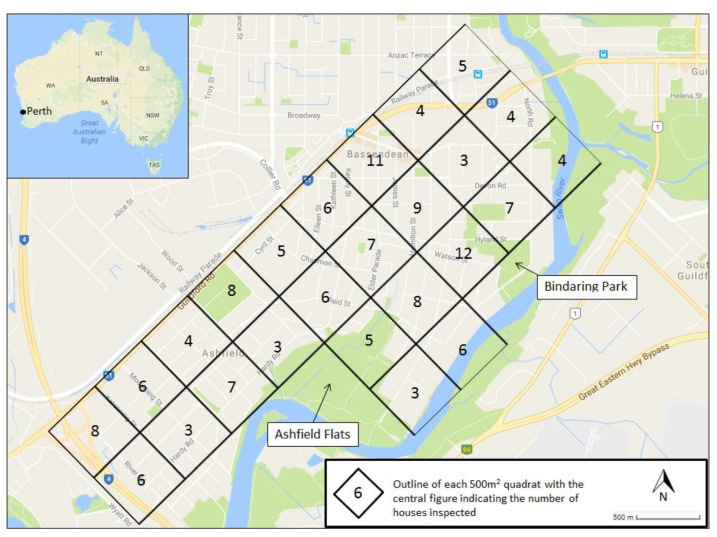
The study area of the Town of Bassendean, Perth Western Australia divided into 25 quadrants. Numbers indicate the randomly generated number of households to be interviewed within each quadrat to obtain KAP (knowledge, attitudes, and practices) data for this survey.

To facilitate data collection, the study area was initially divided into 25 broadly equal-sized quadrants or strata, each comprising approximately 500 m squared (Figure 1). The study area was bordered by the Tonkin Highway to the west, Guildford Road to the north, and the Swan River along the south and east of the study area. A total of 150 households drawn from 25 quadrats were surveyed.

### 2.2. House Selection

Within quadrants, the study plan specified the selection of only one house and resident in any street. Field staff started their selection in each quadrant as closely as practical to its central point, taking into account the constraints of urban design (e.g., sometimes parks were located in the central area). Where households were vacant at the time of interview, the researchers progressed to each adjacent house until they succeeded in recruiting a willing respondent. Once they had conducted an interview, they proceeded to a parallel or abutting street in the quadrant, and applied the same technique. At times, depending on recruitment efforts and the quota for the quadrant, more than one house/respondent was ultimately selected in the same street for interview/inspection. The minimum distance between any two houses in the sample was approximately 200 m.

### 2.3. Questionnaire

The study questionnaire was designed to assess mosquito-related knowledge, attitudes, and practices of respondents. A total of nine questions were put to residents, including both open- and close-ended items. The questionnaire design drew on both the literature and the opinions of expert local stakeholders, and was progressively refined through iterations of review and revision by subject experts, and subsequently, by piloting. Questionnaire design was based on a time-limit of 10–15 min, which was deemed likely to be an acceptable degree of intrusion for householders. There were nine KAP items in the questionnaire, with some multi-component items. Informed consent was obtained from all respondents and only adults were interviewed. Demographic information gathered in the questionnaire included the respondent’s age, gender, home ownership status, period of residence, and the number of respondents living in the household.

### 2.4. Mosquito Abundance

A systematic survey of all 150 residential backyards of those who participated in the questionnaire was carried out, to determine the frequency of backyard container-inhabiting mosquito species. Mosquito larvae were collected from each container, into separate vials, which were labeled with the container type and container number within the surveyed residential lot. All mosquito larvae were successfully reared to adults and identified using taxonomic keys and stereo-microscopes.

Further, Encephalitis Vector Surveillance carbon dioxide (EVS CO_2_) traps were deployed within one backyard of each of the quadrants for a twelve-hour period (overnight), to document the abundance and species composition of mosquitoes within residential backyards. Further, two additional EVS CO_2_ traps were deployed every three weeks at Ashfield Flats and Bindaring Park, to document the mosquito fauna at the natural breeding sites (a total of six collections over the study period). Mosquito abundance was averaged per trap night, for comparison between residential lots (total of 25 traps) and the natural breeding sites (six traps at Ashfield Flats and six traps at Bindaring Park).

### 2.5. Geo Coding and Distance Calculation

To calculate the household distances from known public open space, the centre-points of each quadrant and the respective natural mosquito breeding sites (Ashfield Flats or Bindaring Park) were geocoded. Using both sets of data, approximate distances from households to the main open-space breeding sites were calculated, and these data were used in the subsequent spatial analysis of household KAP data.

### 2.6. Data Analysis

Data analysis was undertaken in SPSS version 22 (SPSS Inc., Chicago, IL, USA) and included the calculation of frequencies, chi-square statistics, and *t*-tests.

## 3. Results

### 3.1. Larvae Identification

**Figure 2:**
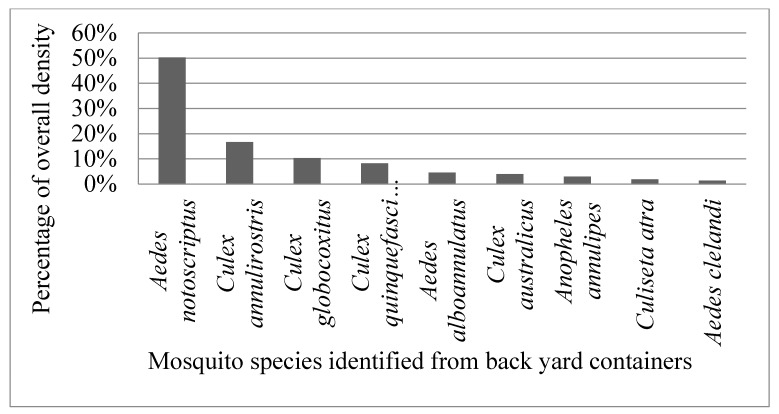
Overall percentage density of mosquitoes from larvae collected from backyard containers across the study area.

A total of 715 mosquito larvae were collected and reared to adults, from 141 of the 150 inspected properties. From the larvae collected, the most commonly reared mosquito was *Aedes notoscriptus*, a known container-inhabiting species accounting for just over 50% of the total larvae collected. This was followed by *Culex annulirostris* Skuse (16.7%), *Culex globocoxitus* Dobrotworsky (10.3%), *Culex quinquefasciatus* Say (8.2%), *Aedes alboannulatus* (Macquart) (4.5%), *Culex australicus* Dobrotworsky & Drummond (4.0%), *Anopheles annulipes* Walker (2.9%), *Culiseta atra (Lee)* (1.9%), and *Aedes clelandi* (Taylor) (1.3%) (Figure 2).

### 3.2. Adult Trapping

**Figure 3:**
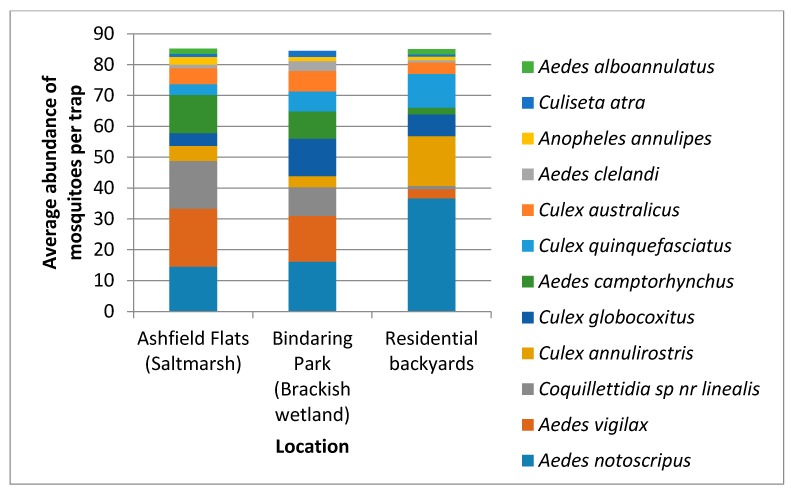
The average abundance of adult mosquitoes collected with EVS CO_2_ traps at Ashfield Flats, Bindaring Park and within residential backyards.

A total of 2126 mosquitoes were collected via EVS CO_2_ traps in residential backyards, with a further 511 mosquitoes from Ashfield Flats and 507 from Bindaring Park. Adult mosquitoes collected with EVS CO_2_ traps reflected the same species distribution as that found with the larval survey. Adult *Aedes notoscriptus* dominated backyards with an average of 36.7 adults per trap. This was followed by *Culex annulirostris* (16.1 adults per trap), *Culex quinquefasciatus* (11), and *Culex globocoxitus* (7). The saltmarsh mosquitoes were only collected in low abundances within residential properties, with *Aedes vigilax* and *Aedes camptorhynchus* only reaching average densities of 3.0 and 2.2 adults per trap, respectively (Figure 3).

### 3.3. Demographics

Respondents (*n* = 150) approximately spanned all age categories in similar proportions (Table 1), and males and females were almost equally represented in the sample (i.e., 51% and 49%, respectively).

Approximately two-in-three respondents (72%) were home owners and had lived in the area for more than five years (66%). Most interviewee households were occupied by couple residents (52%), or adult and child occupants (34%). Only one-in-seven interviewees lived in a single person household (14%).

### 3.4. Knowledge

Seven-in-ten respondents incorrectly reported that either all mosquitoes bite humans (64%), or that only male mosquitoes bite humans (6%). When asked to name mosquito-related diseases they believed that people in Perth were at risk of contracting, most cited RRV (79%), and almost one-in-three mentioned dengue fever (31%). There was also a small proportion who respectively mentioned a range of other mosquito-borne diseases, including MVE (13%), BFV (11%), malaria (9%), and Zika (9%). When asked about the main location of breeding sites or sources of mosquitoes in the local neighborhood, two-in-three respondents mentioned local wetland areas (67%) and the river foreshore (66%). Only one-in-three (33%) indicated that backyards were a primary breeding source.

### 3.5. Attitudes

Attitude questions were generally accompanied by Likert scales, with response options ranging from one to five, with one reflecting “not at all”, while five corresponded with “complete agreement”. Two thirds of respondents felt mosquito problems in the study area were “the same or worse” than in other regions of the Perth, supporting the location’s choice for the study. Notwithstanding this, most respondents (84%) felt the health risks that mosquitoes posed in WA were generally only moderate.

Respondents expressed a limited interest in learning more about how to reduce mosquito problems around their houses, with only one-in-three responding positively to this proposition. The same was true with regards to “doing more in the neighborhood to control local mosquito problems”. This may have been related to there being more than 60% of respondents who believed that mosquito control was “a job for the council and the state government”, rather than for individual households. Notwithstanding this, more than two-in-three respondents agreed that what they did in the neighborhood to control the mosquitoes made a difference to the size of the problem in the broader area. Most study respondents did not see themselves as being more concerned about mosquito problems than other people in their local area, and very few (11%) believed that not enough was being done in their neighborhood to effectively control them.

### 3.6. Practices

With regard to mosquito preventive practices (Table 2), the majority of respondents reported at least sometimes avoiding entertaining outdoors (56%), covering exposed body surfaces (71%), using environmental repellants like outdoor sprays (75%), and using “spray-on” repellants (72%).

Mosquito management measures used in the household by respondents were ascertained as open-ended responses, with the data subsequently summarized into categories reflecting whether responses were preventive-oriented (i.e., future-oriented actions to reduce mosquito breeding potential), or treatment-oriented actions (i.e., addressing the problem once breeding had occurred). Almost one in two respondents (48%) reported taking one or more preventive measures to discourage or reduce mosquito numbers in and around their property. Commonly reported preventive methods were draining stagnant water, cleaning pet water containers and bird baths, planting vegetation that repelled mosquitoes; and introducing fish as larval predators in ponds. One-in-six (17%) respondents also indicated that they used treatment methods such as residual insecticides and fogging. Approximately one-in-three (35%) respondents indicated that they employed no proactive measures to reduce the mosquito numbers in and around their property.

### 3.7. Relationships between Demographics and Mosquito Management Practices

Chi-square analyses of age, gender, and home ownership were undertaken, comparing mosquito management practices by respondents in different categories with these variables. Management measures were categorized as preventive, treatment, or neither. Gender comparisons found that women were significantly more likely to implement preventive practices than males (χ^2^ = 4.54, *p* < 0.05), and less likely to do nothing (χ^2^ = 5.48, *p* < 0.05). There were no other significant differences identified.

### 3.8. Distance from the Breeding Sites and Mosquito Attitudes and Practices

*T*-tests for independent samples were performed, comparing the mean household distance from the known large-scale breeding sites within the study area (i.e., Ashfield Flats and Bindaring Park), across groups defined by mosquito-related attitudes and practices. A significant difference was found in the mean distance from breeding sites across groups defined by the perception of mosquito problems (t = 3.72, df = 146, *p* < 0.05). In this case, those in the group that perceived local problems as being the same or less than other areas of the city, lived an average of approximately 0.5 km further away from the breeding sites. Similarly, a significant difference in the distance from breeding sites was found across groups defined by perceived personal health risk associated with mosquitoes (t = 3.72, df = 146, *p* < 0.05). Once again, lower perceived problems were associated with a greater distance.

With respect to mosquito prevention and treatment practices, groups defined according to whether they did nothing versus doing something, were significantly different in terms of the distance from breeding sites, with distance being associated with inaction (t = 3.10, df = 148, *p* < 0.05). Likewise, those more likely to be practicing preventive measures were significantly closer to breeding areas (t = 2.01, df = 148, *p* < 0.05). The propensity to use mosquito treatment measures was not associated with the distance from breeding areas (t = 0.46, df = 148, *p* > 0.05).

## 4. Discussion

This study used a baseline knowledge, attitudes, and practices (KAP) survey to assess the role of cultural control of mosquitoes amongst a group of residents of the Town of Bassendean, Perth, Western Australia. Such surveys are widely used to collect information across socio-economic groups and socio-cultural communities, to examine health issues and needs [14,15,16].

Successful mosquito management is reliant on an integrated approach. Four basic principles can be used to reduce mosquito breeding, including physical (modification of the environment), chemical, biological, and cultural control methods. Local government authorities in the Perth region have relied heavily on two of these principles. First, physical control or modification of the environment has been pursued through the drainage of wetlands, backfilling of low-lying land, and maintenance of drainage lines and urban drainage systems. Second, through the use of chemical control in high-mosquito-breeding environments like the current study site, through the use of S-methoprene, an insect growth regulator, or Bacillus thuringiensis israelensis (Bti), a bacterium that destroys the gut lining of the mosquito, causing death.

Cultural control, or the modification of human behavior, has been less frequently used by local government authorities, possibly because it has been perceived to be difficult to change human behavior and that there are challenges in reaching some groups. Notwithstanding this, because there is little legislation for mosquito control on private property in Western Australia, cultural control related to breeding “inside the household fence”, needs to be a core part of comprehensive action to reduce mosquitoes in urban areas. Given this, the following summary of findings should provide local authorities interested in designing cultural control activities and programs, with useful points of departure.

### 4.1. Mosquito Knowledge among the Public Seems Limited

In general, knowledge of mosquitoes and mosquito-related disease was poor amongst study respondents, with only 30% correctly identifying that only female mosquitoes bite humans. While a high percentage of respondents were able to identify RRV as a mosquito-borne infection (79%), the second most commonly reported was Dengue virus, an exotic infection which is not transmitted within Western Australia. In contrast, three other infections endemic to Western Australia were poorly identified including MVE (13% of respondents), BFV (11% of respondents), and WNV_KUN_ (0% of respondents). These results are similar to those reported by Potter et al. [13], who also found that RRV was well recognized among Western Australian respondents in their State-wide survey, but that there is a limited awareness of other endemic infections.

Notably, ZIKV, which was not identified by any respondents in the current study prior to January 2016, was subsequently reported following the media attention given to the virus associated with the Olympic Games held in Brazil. Overall, nine percent of study respondents mentioned Zika, which is strongly suggestive of the role that media can play in increasing mosquito-related infection and disease awareness.

Knowledge of mosquito breeding sites among the current study’s respondents also emphasized natural breeding areas like wetlands (67%) and the river foreshore (66%), running counter to Potter et al.’s earlier findings [13], who reported that only 33% of state-wide respondents identified wetlands as breeding sites. This points to the local context being a major influence on resident perspectives of factors contributing to mosquito problems, and demonstrates a need to present public health education messages that are tailored to the neighborhood context.

Importantly, only 33% of respondents in the current study believed that mosquito breeding occurred in local residential lots, which is similar to Western Australian data from a previous state-wide survey (20.8%) [13]. This suggests a need for public education to raise awareness of the potential for backyard breeding in urban areas of Perth to cause nuisance and to play a role in minimizing disease risk from mosquitoes.

### 4.2. Attitudes

The attitudes of study respondents indicated that they were concerned about mosquito-borne disease, with two thirds of respondents believing that mosquito risks were similar or worse than other urban areas within the Perth metropolitan area. This is substantially higher than the 25% recorded by Potter et al. [13], and may be associated with the close proximity to the Swan River and high nuisance levels of mosquitoes associated with the productive mosquito breeding habitat at nearby Ashfield Flats.

Only one third of respondents, however, expressed a willingness to learn more about the prevention of mosquito breeding and transmission of infection, with 60% indicating that the problem was solely the responsibility of the State and local authorities. Unfortunately, neither the State nor Local Government has a legislated mandate for mosquito management on private land, leaving education as a key strategy in controlling breeding capacity in residential lots.

### 4.3. Practices

Sixty-three per cent of respondents in this study indicated that they wore protective clothing (e.g., long sleeved, loose, and light colored) to avoid mosquito bites. The use of chemicals (including repellents, residual surface sprays, mosquito coils, and candles) was less frequently reported as a tool for managing mosquito problems. Approximately half of the respondents undertook basic physical control measures to reduce mosquito breeding, including emptying stagnant water, cleaning pet water bowls, or the use of fly screens. This finding accords with a Malaysian study, in which only four-in-ten (39%) respondents reported keeping their yards clear of debris and mosquito breeding containers, in [17]. Further education of the public seems to be required to ensure these low-cost or free physical control methods are more widely used to reduce the impact of mosquito-related nuisance and disease in the State.

The current study also identified that one-in-three respondents (35%) took no proactive measures to reduce mosquito numbers in and around their property, which reflects the need for persistent awareness-raising efforts in relation to mosquito control, to address what appears to be a substantial risk for widespread complacency.

### 4.4. Spatial Relationship

Analysis of the responses according to the proximity to productive mosquito breeding sites suggested that as the distance increased, concern about mosquitoes and mosquito-borne diseases declined, as did active treatments, including chemical use. This is not surprising, and accords with a number of other studies conducted in Western Australia, particularly in the Peel, Geographe, and Leschenault regions, south of Perth [10,18,19]. Regardless, public education campaigns are needed to educate the community as a whole, to reduce the incidents of mosquitoes within on-site infrastructure.

### 4.5. Study Limitations

When conducting population surveys, there is always the potential for sample bias. The challenge in this study was that reliable denominator data were not available for the study site, because it formed a portion of the statistical area. Notwithstanding this limitation to claims of representativeness, the study sample comprised a 50:50 ratio between males and females and a similar number of responses from each of the three age categories (18–35, 35–55 and 56+), and a mix of home owners (72%) and renters (28%). Because the survey was only performed in one local government of the Perth metropolitan region, different results may have been found if the survey was performed across multiple Local Government areas, including those at a greater distance from the Swan River. Additionally, while the survey was conducted during the most conducive time of year for high mosquito populations, the climatic conditions were not conducive to mosquito breeding during the summer of 2015–2016. Conducting the survey across multiple years may detect variation in responses, in direct relation to mosquito populations at the time of the survey.

### 4.6. Community Awareness Campaigns

The high percentage of *Ae. notoscriptus* found across residential backyards (50% of all mosquito larvae collected) indicates that further education of residents is required, to reduce mosquito nuisance around the home. Since discussion of the results of the study with the Local Government Authority, a number of measures have been implemented to increase awareness and participation of the community. Initially, flyers and information brochures were distributed to residents with annual rates notices in August, prior to the start of the mosquito season (September), to notify residents of their responsibility and to request the removal of potential backyard breeding containers. In addition, the Environmental Health Officer has written a number of articles for publication in the local “Bassendean Briefings” booklets that are distributed by the Local Government. Further, the State Government has funded a “Fight the Bite” media campaign, providing information and advertising at the local outdoor cinema during the summer period.

It has been recognized that further initiatives are required to raise awareness, and reduce mosquito nuisance and potential disease risks within the Town. Currently, consideration is being given to the development of a Facebook page, and the role of a curriculum-based school training package that has been developed in consultation with the Department of Health, Western Australia and the South East Regional Centre for Urban Landcare (SERCUL). It has been recognized that communication is required across multiple age groups (including school children who may take key messages home and parents). Other strategies, including booths at council events and the role of a “clean up your yard” day or week, could also be useful for refocusing the public’s attention on this ongoing public health concern. Regardless, a communication strategy is required to ensure the community are reminded of their roles and responsibilities in regards to mosquito breeding on private property.

## 5. Conclusions

Currently, the Western Australian State Department of Health has a role to play in monitoring mosquito-related disease notifications and virus detection through mosquito surveillance, and actively uses mass media campaigns and media statements to alert the public to infection and disease risks. The State Department of Health works collaboratively with Local Governments across Western Australia through a state-wide Contiguous Local Authority Group (CLAG) funding scheme, to provide resources for the management of mosquito populations in high-risk areas. Local Government Environmental Health Officers are tasked with monitoring mosquito populations and taking control measures to reduce both nuisance and disease risks. However, the current legislation only allows these activities on public property and it is the role of private residents to manage mosquito breeding around their homes. The results of this survey have highlighted a lack of both knowledge and willingness of residents to reduce mosquito breeding around their homes. A public health media campaign has since been initiated by the Town of Bassendean to increase knowledge, and improve attitudes and practices of their residents, for reducing backyard breeding.

## Figures and Tables

**Table 1:** Age composition of respondents to the KAP survey.

Age Group	% (*n* = 150)
18–35 years	26
36–55 years	39
56+ years	35

**Table 2:** Preventive practices used to avoid mosquitoes around the home.

Preventive Practices	Never (%)	Sometimes (%)	Often (%)	Always (%)
Avoid outdoor entertaining	44	35	15	6
Wear clothing and footwear that minimizes the chances of being bitten	29	43	20	8
Stay indoors during the late afternoon and evenings	36	39	18	7
Avoid gardening	62	28	6	4
Use outdoor sprays, mosquito coils, candles or lamps	25	38	25	12
Use spray-on repellents	28	33	24	15
